# The Influence of Parental Psychopathology on Offspring Suicidal Behavior across the Lifespan

**DOI:** 10.1371/journal.pone.0134970

**Published:** 2015-07-31

**Authors:** Geilson Lima Santana, Bruno Mendonca Coelho, Guilherme Borges, Maria Carmen Viana, Yuan Pang Wang, Laura Helena Andrade

**Affiliations:** 1 Section of Psychiatric Epidemiology – LIM 23, Department and Institute of Psychiatry, University of Sao Paulo Medical School, Sao Paulo, Brazil; 2 National Institute of Psychiatry and Metropolitan Autonomous University, Mexico City, Mexico; 3 Department of Social Medicine and Post-Graduate Program in Public Health, Federal University of Espirito Santo, Vitoria, Brazil; The George Institute for Global Health, INDIA

## Abstract

Suicide tends to occur in families, and parental psychopathology has been linked to offspring suicidal behaviors. This study explores the influence of parental mental disorders across the lifespan. Data are from the Sao Paulo Megacity Mental Health Survey, a cross-sectional household study with a representative sample of the adult population living in the Sao Paulo Metropolitan Area, Brazil (N=2,942). Survival models examined bivariate and multivariate associations between a range of parental disorders and offspring suicidality. After controlling for comorbidity, number of mental disorders and offspring psychopathology, we found that parental psychopathology influences suicidal behaviors throughout most part of the life cycle, from childhood until young adult years. Generalized anxiety disorder (GAD) and antisocial personality were associated with offspring suicidal ideation (OR 1.8 and 1.9, respectively), panic and GAD predicted suicidal attempts (OR 2.3 and 2.7, respectively), and panic was related to the transition from ideation to attempts (OR 2.7). Although noticed in many different stages of the lifespan, this influence is most evident during adolescence. In this period, depression and antisocial personality increased the odds of suicidal ideation (OR 5.1 and 3.2, respectively), and depression, panic disorder, GAD and substance abuse predicted suicidal attempts (OR varying from 1.7 to 3.8). In short, parental disorders characterized by impulsive-aggression and anxiety-agitation were the main predictors of offspring suicidality across the lifespan. This clinically relevant intergenerational transmission of suicide risk was independent of offspring mental disorders, and this underscores the need for a family approach to psychopathology.

## Introduction

Suicide is a major public health concern. Each year, over 800,000 people die by intentional self-harm, making it the fifteenth leading cause of death globally [1]. Suicide happens across the lifespan, and in the last decades, rates have risen among adolescents and young adults [2]. It is now the second most common cause of mortality in the 15–29 year-olds, accounting for 8.5% of deaths in this age group [1].

Suicide may be best conceived as the most severe outcome of a broader range of behaviors, which also include ideation, plans and attempts. Nonfatal suicidal behavior is 10 to 20 times more frequent than suicide [3] and constitutes an important risk factor for later death by intentional self-harm [4]. Understanding the predictors of suicidal ideation, plans and attempts may thus help reduce suicide occurrence.

Suicidality tends to run in families [5–7], but the mechanisms for this intergenerational transmission remain largely unknown. Part of the risk may be explained by a family history of mental disorders, and parental psychopathology has been linked to offspring suicidal behaviors [8–15]. In the World Mental Health Survey Initiative (WMH), diverse parental disorders were shown to increase the odds of suicidal ideation in the offspring; parental generalized anxiety predicted the onset, and depression, the persistence of suicidal plans; and antisocial personality and anxiety disorders predicted the transition from ideation to attempts [16]. However, an important gap remains in the literature: it is unclear what is the influence of parental psychopathology on different stages of the life cycle, since most studies looked specifically to teenage or adulthood suicidality or reported results considering the lifespan as a whole [8–18].

This study aims to explore the association between parental psychopathology and offspring suicidality in specific lifecycle periods, examining when this influence is most prominent, its duration, and the primary predictors in each lifecycle stage. We examined these associations in the Sao Paulo Metropolitan Area, Brazil. According to the World Health Organization (WHO), 75% of suicides occur in low- and middle-income countries, and in Brazil, mortality rates have increased 10.4% between 2000 and 2012 [1]. However, data on suicide from developing countries are scarce [19]. There are marked differences in suicidal behavior between developed and less-developed nations [20], and our data may help shed light into specific aspects of the trajectories to suicide in developing regions.

## Materials and Methods

### Sample

Data are from the Sao Paulo Megacity Mental Health Survey (SPMHS), a cross-sectional household study of psychiatric morbidity with a representative sample of the adult population living in the Sao Paulo Metropolitan Area, Brazil [21]. This region is comprised of the city of Sao Paulo and its 38 surrounding municipalities, and at the time of data collection (2005–2007), 11 million inhabitants were 18 years or older [22].

Respondents were selected by means of a stratified, multistage area probability sample of households. In each household, the interviewer obtained a list of all residents, with information on age, gender and family relationship to the informant. This list was then sorted by gender and inverse order of age, and the eligible respondents were identified, i.e., those who were 18 years or older, Portuguese-speaking and without any disability or handicap that would impair their ability to participate in the study. One resident was then randomly selected by means of a Kish grid, a probabilistic method for selecting household respondents from a table of random numbers [23]. In addition, in a random 20% sample of households where the selected respondent was married or living as married, the spouse was identified and selected for interview. A total of 5,037 subjects were evaluated, with a global response rate of 81.3%.

The interview was administered in two parts. Part I assessed the presence of core mental disorders and suicidal behaviors, as well as sociodemographic variables, while Part II assessed a few non-core diagnoses and potential correlates of psychopathology and suicidality, such as parental mental disorders. Part I was administered to all 5,037 respondents, while Part II was administered to 2,942 subjects, including all of those who met lifetime criteria for Part I disorders and a probability subsample of other respondents. Data were weighted to adjust for the under-sampling of Part II non-cases and to adjust for residual discrepancies between sample and population distributions on a range of socio-demographic variables. Part II respondents are the focus of the current report. Detailed descriptions of sampling and weighting methods are presented elsewhere [24].

The Ethical and Research Committee of the University of Sao Paulo Medical School approved the SPMHS procedures. Respondents were interviewed only after informed written consent was obtained and total confidentiality was assured.

### Measures

Respondents were assessed using the World Mental Health Survey Composite International Diagnostic Interview (WMH-CIDI) [25], a fully structured lay interview that yields diagnoses according to DSM-IV criteria. The instrument was translated and adapted to the Brazilian-Portuguese language [26], and trained professional interviewers carried out face-to-face interviews.

#### Suicidal behavior

The suicidality module of the WMH-CIDI included assessment of the lifetime occurrence and age-of-onset of suicidal ideation, plan and attempt. Data were provided on the following suicidal behaviors: *suicidal ideation* in the total sample; *suicidal attempt* in the total sample; *suicidal plan* among ideators; and *suicidal attempt* among ideators.

#### Mental disorders in the offspring

Respondents were assessed with the WMH-CIDI for the lifetime occurrence of DSM-IV *mood* (major depressive disorder, dysthymia, bipolar disorder I or II), *anxiety* (panic disorder, agoraphobia without panic disorder, specific phobia, social phobia, GAD, post-traumatic stress disorder, obsessive-compulsive disorder, adult separation anxiety), *impulse-control* (intermittent explosive disorder, oppositional-defiant disorder, conduct disorder, attention deficit/hyperactivity disorder) and *substance use* (alcohol and drug abuse or dependence) *disorders*. Organic exclusion rules were used in making diagnoses.

#### Parental psychopathology

Parental psychopathology was assessed with the expanded version of the Family History Research Diagnostic Criteria Interview [27,28]. Five different forms of parental psychopathology during respondent’s childhood were considered in the present report: major depression, panic disorder, generalized anxiety disorder (GAD), substance abuse and antisocial personality disorder. Parental suicidal behavior was also assessed, but the positive answers were too few to allow analysis and were thus excluded from this report. The original version of the instrument also evaluates other categories of parental psychopathology, but this study included only the main predictors of offspring suicidality according to previous research [5,8,9,11,12,15,29–34]. This report is part of a larger epidemiologic survey, and this selection was done to limit the extension of the interview and minimize respondent burden.

A parental psychiatric disorder was rated present if the respondent gave a positive response to questions on the core symptoms of that particular disorder occurring in the mother or the father. Prior analyses showed that the relationship between parental mental disorders and offspring suicidality was independent of whether the mother, the father or both were affected [16] and here we thus ignore this distinction.

### Data Analysis

The associations between parental psychopathology and offspring suicidality were estimated in a series of bivariate and multivariate discrete-time survival models, with person-year as the unit of analysis [35]. This methodology is defined in greater detail elsewhere [16]. Briefly, we used bivariate models in which each type of parental psychopathology was considered individually in predicting offspring suicidality. Next, we estimated multivariate models that considered all parental disorders simultaneously in predicting each suicidal behavior. We also fitted multivariate models testing the relationship between number of parental disorders and likelihood of each suicidal behavior. We then estimated a series of multivariate models in which both the type and number of parental disorders were included in order to examine the unique contribution of both specific forms of parental psychopathology and the total number of parental disorders. These final models also controlled for mental disorders in the offspring. Finally we used a series of multivariate models to analyze the association of type and number of parental disorders and each suicidal behavior across the lifespan. All multivariate models controlled for person-years (in intervals varying from 1 up to 5 person-years), demographics (sex, age, time-varying education and time-varying marital status), and also the significant interaction terms from the demographics. Since education and marital status may have changed across the observation framework, they were regarded as time-varying covariates [35].

To adjust for the weighting and clustering of the stratified multistage sample design, standard errors and significance tests were estimated using the Taylor series method [36] using SUDAAN software [37]. Multivariate significance was evaluated using Wald χ^2^ tests based on design-corrected coefficient variance-covariance matrices. All significance tests were evaluated using .05 level two-sided tests.

To aid interpretation, survival coefficients were converted to odds ratios (ORs) and their standard errors were generated by exponentiating the survival coefficients.

## Results

### Lifetime prevalence of offspring suicidal behaviors and parental psychopathology

The lifetime prevalence of suicidal ideation, plans and attempts was, respectively, 13.84%, 4.95% and 3.39%. Among those with suicidal ideation (n = 585), more than a third also presented suicidal plans; and among those with suicidal plans, 47% attempted suicide. Women exhibited higher rates of all these suicidal outcomes.

As shown in Table 1, the prevalence of parental psychopathology was higher among those presenting suicidal ideation or attempts, being panic disorder, GAD, and substance abuse the most prevalent diagnoses. Likewise, the prevalence of parental panic disorder was higher among those who evolved from ideation to plans. There was a dose-response relationship between the severity of suicidal behavior and the prevalence of parental psychopathology, and comorbidity was more common than single disorders in the ideation and attempts subgroups. As indicated previously, the few positive cases of parental suicidal behavior precluded further analyses. In brief, the prevalence estimates of maternal and paternal suicide attempt were 3.17% and 0.97%, respectively (not shown).

**Table 1:** Weighted prevalence of parental psychopathology in relation to offspring lifetime suicidality.

Parental psychopa-thology	Total sample						Among ideators					
	Ideation	No ideation	χ^2^	Attempts	No attempts	χ^2^	Plans	No plans	χ^2^	Attempts	No attempts	χ^2^
	% (SE)	% (SE)		% (SE)	% (SE)		% (SE)	% (SE)		% (SE)	% (SE)	
**Depres-sion** ^1^	9.0% (1.2)	3.3% (0.5)	12.6*	13.2% (2.2)	3.8% (0.4)	13.5*	12.0% (2.5)	7.4% (1.4)	2.4	13.2% (2.2)	7.7% (1.5)	4.5*
**Panic disorder** ^1^	25.0% (2.3)	14.4% (1.2)	16.6*	35.7% (5.1)	15.2% (1.1)	12.4*	31.9% (3.3)	21.1% (2.8)	6.5*	35.7% (5.1)	21.5% (2.6)	4.9*
**GAD** ^1^	13.3% (1.8)	4.9% (0.7)	15.6*	21.5% (4.0)	5.6% (0.6)	13.2*	18.2% (3.7)	10.6% (2.1)	3.2	21.5% 4.0)	10.6% (1.9)	6.1*
**Substance abuse** ^1^	14.5% (1.9)	9.0% (0.9)	8.7*	21.1% (4.4)	9.3% (0.8)	6.8*	14.4% (3.5)	14.5% (2.3)	0.0	21.1% (4.4)	12.3% (1.8)	3.5
**ASP** ^1^	8.4% (1.7)	3.1% (0.4)	9.1*	9.2% (2.3)	3.7% (0.5)	4.8*	8.2% (1.7)	8.5% (2.2)	0.0	9.2% (2.3)	8.1% (2.0)	0.1
**Number of parental disorders**												
**1**	10.2% (1.1)	6.3% (0.8)	12.7*	12.8% (2.5)	6.6% (0.8)	5.1*	16.8% (3.1)	6.6% (1.6)	5.6*	12.8% (2.5)	9.4% (1.5)	1.1
**2**	11.7% (1.8)	8.4% (1.0)	2.2	13.9% (2.9)	8.7% (0.9)	2.8	13.0% (1.7)	11.1% (2.5)	0.4	13.9% (2.9)	11.0% (2.0)	0.8
**3+**	12.0% (1.5)	4.0% (0.5)	26.9*	19.5% (4.1)	4.6% (0.5)	11.0*	13.8% (2.9)	11.0% (2.2)	0.5	19.5% (4.1)	9.6% (1.6)	4.2

GAD: Generalized Anxiety Disorder; ASP: Antisocial Personality Disorder; SE: Standard Error

^1^ One or both parents

* Significant at the .05 level, two-sided test

### Associations between parental psychopathology and offspring suicidality

Bivariate survival models revealed that each form of parental psychopathology was significantly associated with increased odds of subsequent first onset of both suicidal ideation and attempts (OR varying from 1.5 to 3.4). However, in the multivariate models, only GAD and antisocial personality remained as significant predictors of suicidal ideation (OR 1.7 and 1.8, respectively); and only panic disorder and GAD predicted suicidal attempts (OR 1.8 and 2.1, respectively). No parental disorder was associated with suicidal plans or attempts among ideators (Table 2).

**Table 2:** Associations between parental psychopathology and offspring lifetime suicidality^a^.

Parental psychopa-thology	Total sample				Among ideators			
	Ideation^b^		Attempts^c^		Plans^d^		Attempts^e^	
	Bivariate OR	Multivariate OR	Bivariate OR	Multivariate OR	Bivariate OR	Multivariate OR	Bivariate OR	Multivariate OR
	(95% CI)	(95% CI)	(95% CI)	(95% CI)	(95% CI)	(95% CI)	(95% CI)	(95% CI)
**Depression** ^1^	**2.5 (1.6–3.7)** *	1.4 (0.9–2.3)	**3.1 (2.0–4.7)** *	1.3 (0.8–2.0)	1.0 (0.5–2.2)	0.7 (0.4–1.3)	0.8 (0.4–1.9)	0.7 (0.4–1.3)
**Panic** ^1^	**2.0 (1.3–3.0)** *	1.3 (0.9–2.0)	**3.0 (1.9–4.7)** *	**1.8 (1.0–3.4)** *	1.7 (0.8–3.4)	1.8 (0.8–3.8)	1.5 (1.0–2.5)	1.8 (0.8–3.8)
**GAD** ^1^	**2.5 (1.7–3.7)** *	**1.7 (1.0–2.8)** *	**3.4 (2.2–5.3)** *	**2.1 (1.0–4.2)** *	1.4 (0.7–2.9)	1.4 (0.7–2.8)	0.7 (0.3–1.6)	1.4 (0.7–2.8)
**Substance abuse** ^1^	**1.5 (1.1–2.0)** *	1.1 (0.8–1.5)	**1.7 (1.0–2.7)** *	1.2 (0.7–1.9)	0.9 (0.4–1.8)	0.8 (0.4–1.6)	0.7 (0.3–1.7)	0.8 (0.4–1.6)
**ASP** ^1^	**2.2 (1.5–3.4)** *	**1.8 (1.1–2.8)** *	**1.9 (1.0–3.7)** *	1.3 (0.6–2.6)	1.0 (0.5–1.9)	0.9 (0.5–1.6)	0.9 (0.3–2.3)	0.9 (0.5–1.6)
**Number of parental disorders**								
	**Multivariate OR**		**Multivariate OR**		**Multivariate OR**		**Multivariate OR**	
	**(95% CI)**		**(95% CI)**		**(95% CI)**		**(95% CI)**	
**1**	**1.4 (1.1–1.9)** *		**2.3 (1.5–3.5)** *		1.5 (0.6–3.6)		1.2 (0.8–1.9)	
**2**	**2.7 (1.8–4.1)** *		**3.1 (2.0–4.8)** *		1.3 (0.7–2.5)		0.8 (0.4–1.4)	
**3+**	**2.7 (1.7–4.3)** *		**—**		**—**		**—**	

GAD: Generalized Anxiety Disorder; ASP: Antisocial Personality Disorder; OR (95% CI): Odds ratio (95% Confidence Interval)

^1^ One or both parents

* Significant at the .05 level, two-sided test

—Cell size too small for analyses

^**a**^ Models control for person-years (1 to 5 person-year intervals) and demographics (sex, age, time-varying education and time-varying marital status), and also the significant interaction terms from the demographics. Details in the following footnotes.

^**b**^ Models control for person-years (1 to 5 person-year intervals), demographics (sex, age, time-varying education, time-varying marital status), and also the interaction between person-year intervals and sex, age, education, marital status.

^**c**^ Models control for person-years (1 to 5 person-year intervals), demographics (sex, age, time-varying education, time-varying marital status), and also the interaction between person-year intervals and sex, age, education, marital status.

^**d**^ Models control for person-years (1 to 5 person-year intervals), demographics (sex, age, time-varying education, time-varying marital status), and also the interaction between person-year intervals and age, education.

^**e**^ Models control for person-years (1 to 5 person-year intervals), demographics (sex, age, time-varying education, time-varying marital status), and also the interaction between person-year intervals and sex, age, education.

There was a dose-response relationship between the number of parental disorders and suicidal ideation and attempts in the total sample: the greater the number of diagnoses, the greater the prevalence of these suicidal behaviors. There was no significant influence of the number of parental disorders on suicidal plans or attempts among ideators.

As seen on Table 3, both type and number of parental disorders were included simultaneously in the final multivariate model, with control for offspring psychopathology. GAD and antisocial personality predicted suicidal ideation (OR 1.8 and 1.9, respectively); parental panic disorder and GAD remained associated with suicidal attempts in the total sample (OR 2.3 and 2.7, respectively); and panic disorder was a significant predictor of transition from ideation to attempts (OR 2.7). The association between number of parental disorders and offspring suicidality did not remain significant in the final model.

**Table 3:** Final multivariate model for associations between type and number of parental disorders and offspring lifetime suicidality^a^.

Parental psychopathology	Total sample		Among ideators	
	Ideation^b^	Attempts^c^	Plans^d^	Attempts^e^
	OR (95% CI)	OR (95% CI)	OR (95% CI)	OR (95% CI)
**Depression** ^1^	1.7 (0.9–3.1)	1.5 (0.9–2.6)	0.7 (0.4–1.4)	0.8 (0.3–2.4)
**Panic disorder** ^1^	1.4 (0.9–2.3)	**2.3 (1.1–4.6)** *	2.1 (0.9–4.9)	**2.7 (1.2–5.8)** *
**GAD** ^1^	**1.8 (1.1–3.1)** *	**2.7 (1.3–5.5)** *	1.7 (0.5–5.9)	0.9 (0.3–2.6)
**Substance abuse** ^1^	1.1 (0.8–1.6)	1.3 (0.8–2.2)	0.8 (0.4–2.0)	0.9 (0.5–1.9)
**ASP** ^1^	**1.9 (1.1–3.1)** *	1.5 (0.7–3.3)	1.1 (0.5–2.2)	1.2 (0.4–3.4)
**Number of parental disorders**				
**2**	1.2 (0.8–1.9)	0.5 (0.2–1.0)	0.7 (0.1–4.0)	0.5 (0.1–1.7)
**3+**	0.6 (0.3–1.3)	**—**	**—**	**—**

GAD: Generalized Anxiety Disorder; ASP: Antisocial Personality Disorder; OR (95% CI): Odds ratio (95% Confidence Interval).

^1^ One or both parents

* Significant at the .05 level, two-sided test.

—Cell size too small for analyses.

^**a**^ Models control for person-years (1 to 5 person-year intervals) and demographics (sex, age, time-varying education and time-varying marital status), and also the significant interaction terms from the demographics, as well as for mental disorders in the offspring. Details in the following footnotes.

^**b**^ Models control for person-years (1 to 5 person-year intervals), demographics (sex, age, time-varying education, time-varying marital status), and also the interaction between person-year intervals and sex, age, education, marital status, as well as for mental disorders in the offspring.

^**c**^ Models control for person-years (1 to 5 person-year intervals), demographics (sex, age, time-varying education, time-varying marital status), and also the interaction between person-year intervals and sex, age, education, marital status, as well as for mental disorders in the offspring.

^**d**^ Models control for person-years (1 to 5 person-year intervals), demographics (sex, age, time-varying education, time-varying marital status), and also the interaction between person-year intervals and age, education, as well as for mental disorders in the offspring.

^**e**^ Models control for person-years (1 to 5 person-year intervals), demographics (sex, age, time-varying education, time-varying marital status), and also the interaction between person-year intervals and sex, age, education, as well as for mental disorders in the offspring.

### Associations between parental psychopathology and offspring suicidality across the lifespan

Next step, the associations between parental psychopathology and offspring suicidality were explored during childhood (ages 3–12 years) and adolescence (ages 13–19 years), as well as their long-term effects up to young adulthood (ages 20–29 years) and late adulthood (ages 30 years and over) (Table 4).

**Table 4:** Associations between parental psychopathology and offspring suicidality across the lifespan^a^.

	Childhood		Adolescence		Young adulthood		Late adulthood	
	Ideation^b^	Attempts^c^	Ideation^b^	Attempts^c^	Ideation^b^	Attempts^c^	Ideation^b^	Attempts^c^
	OR (95% CI)	OR (95% CI)	OR (95% CI)	OR (95% CI)	OR (95% CI)	OR (95% CI)	OR (95% CI)	OR (95% CI)
**Depression** ^1^	0.3 (0.0–3.0)	0.3 (0.0–7.9)	**5.1 (1.9–13.7)** *	**3.2 (1.3–8.0)** *	1.2 (0.5–2.8)	1.3 (0.4–4.6)	0.6 (0.2–1.6)	0.5 (0.2–1.5)
**Panic** ^1^	0.2 (0.0–1.6)	2.2 (0.7–6.7)	2.8 (0.9–8.7)	**3.8 (1.0–14.7)** *	0.9 (0.4–2.2)	1.7 (0.4–7.6)	1.0 (0.5–2.2)	1.0 (0.3–3.3)
**GAD** ^1^	2.3 (0.5–11.6)	**64.7 (8.0–521.7)** *	2.2 (0.9–5.9)	**3.3 (1.1–9.9)** *	**3.6(1.5–8.2)** *	**3.7 (1.0–13.1)** *	0.6 (0.2–1.5)	0.6 (0.2–2.3)
**Substance abuse** ^1^	0.7 (0.1–3.6)	**0.1 (0.0–0.1)** *	1.1 (0.5–2.2)	**1.7 (1.0–3.0)** *	1.3 (0.7–2.1)	1.0 (0.3–3.3)	1.0 (0.5–2.0)	1.4 (0.4–4.9)
**APD** ^1^	0.2 (0.0–3.1)	**0.1 (0.0–0.3)** *	**3.2 (1.4–7.1)** *	1.7 (0.5–5.5)	1.9 (0.6–5.8)	1.4 (0.4–5.1)	1.1 (0.5–2.6)	1.5 (0.4–5.5)

GAD: Generalized Anxiety Disorder; ASP: Antisocial Personality Disorder; OR (95% CI): Odds ratio (95% Confidence Interval).

^1^ One or both parents

* Significant at the .05 level, two-sided test.

^**a**^ Models control for person-years (1 to 5 person-year intervals) and demographics (sex, age, time-varying education and time-varying marital status), and also the significant interaction terms from the demographics, as well as for mental disorders in the offspring. Details in the following footnotes.

^**b**^ Models control for person-years (1 to 5 person-year intervals), demographics (sex, age, time-varying education, time-varying marital status), and also the interaction between person-year intervals and sex, age, education, marital status, as well as for mental disorders in the offspring.

^**c**^ Models control for person-years (1 to 5 person-year intervals), demographics (sex, age, time-varying education, time-varying marital status), and also the interaction between person-year intervals and sex, age, education, marital status, as well as for mental disorders in the offspring.

The most evident influences of parental psychopathology on offspring suicidality could be found during adolescence: Parental depression and antisocial personality increased the odds of suicidal ideation (OR 5.1 and 3.2, respectively); and parental depression, panic disorder, GAD and substance abuse predicted suicidal attempts (OR varying from 1.7 to 3.8). During adulthood, parental GAD was associated with suicidal ideation and attempts (OR 3.6 and 3.7, respectively). No effect of parental psychopathology on offspring suicidality was observed in later adulthood.

One must note that during childhood, data indicate that parental GAD increased the odds of suicidal attempts, and that substance abuse and antisocial personality tended to reduce the odds of this behavior. However, the limited number of cases precludes any firm statements.

## Discussion

The key results of this study are that parental psychopathology is significantly associated with suicidality in the offspring, and this effect is most evident during adolescence. Comorbidity is a common occurrence, and after controlling for the number and type of disorders and offspring psychopathology, parental anxiety (GAD and panic) and antisocial personality remained related to suicidal ideation and attempts. During adolescence, parental depression also emerged as a significant predictor of suicidal behavior.

These findings are similar to those of the pooled analysis of the World Mental Health Survey Initiative. However, in the WMH, all parental disorders examined were associated with offspring suicidality [16], while in the current study, only GAD and antisocial personality were related to ideation; and only panic and GAD were associated with suicidal attempts. Moreover, the transition from ideation to attempts was predicted, in the former study, by parental panic, GAD, substance abuse and antisocial behavior, and in the current analysis, by panic. In this respect, our results are in greater consonance with those found in Mexico [17], Nigeria [18] and South Africa [38], which highlighted the role of disorders characterized by impulsive-aggression (antisocial personality) and anxiety-agitation (generalized anxiety and panic disorder). This may be a consequence of the greater similarities of these populations, all originated from developing countries, in contrast to the diverse populations included in the WMH analysis.

Suicidal behaviors are complex phenomena and result from the interactions of biological, psychological, family, sociocultural and environmental risk factors [39]. As stated before, there tends to be a familial aggregation of suicidality [5–7], but the mechanisms for this intergenerational transmission are not fully understood. It cannot be explained by the transmission of mental disorders, since the associations found in this study remained significant even after controlling for offspring diagnoses. A more likely explanation is the transmission of intermediate phenotypes, such as subclinical psychopathological traits. The association between parental antisocial personality and offspring suicidal behaviors may be partially explained by the transmission of cluster B traits and impulsive aggression, that is, the tendency to react to disappointment or frustration with impulsive, often aggressive responses [31,40]. Research has also shown that the familial transmission of suicidal behaviors may be mediated by neuroticism [41], and this may explain the association between parental anxiety and offspring suicidality. One must consider as well the participation of environmental mechanisms, such as the exposure to stressors during childhood and the intergenerational transmission of adverse family environments [42]. Moreover, the influence of parental panic on the transition from ideation to attempts may be related to high limbic activation, sympathetic arousal and “fight or flight” responses [16,43].

As seen, the influence of parental psychopathology on offspring suicidality is most evident during adolescence and this may be due to the convergence of the aforementioned factors during this life stage. Beyond the influence of inherited psychopathological traits and adverse family environments, teenagers are faced with physical modifications of the body and the brain. This is a period of emotional turmoil and heightened impulsivity, at least partly explained by an imbalance in the development of limbic structures (as the amygdala) in relation to the prefrontal cortex [44]. Furthermore, adolescents must deal with new psychosocial challenges due to changes in family and social roles, identity and interpersonal relationships, resulting in the juxtaposition of stressors of different natures.

The findings presented here must be considered taking into account the limitations presented by this study. All data regarding parental psychopathology and suicidal behavior were retrospectively assessed by offspring self-report. Even though childhood events can be recalled with accuracy [45], the results may have been affected by recall bias. In addition, our analysis didn’t include all potentially relevant parental mental disorders (e.g., schizophrenia and borderline personality), as well as a measure of severity or persistence of parental psychopathology. Moreover, the study may have lacked power to show further associations.

Globally, our findings have important implications for research, clinical practice and health policies. They contribute to a greater understanding of the influence of parental psychopathology on offspring suicidality, and indicate that the transmission of suicidal behaviors is independent of the transmission of mental disorders themselves. Hence, future research must go beyond psychiatric categories and include measures of psychopathological traits and dimensions in order to elucidate the mediators and moderators of this association. The most appropriate design would be a prospective, genetically sensitive cohort to allow differentiation of genetic and environmental influences, with inclusion of other parental mental disorders and criteria of severity and persistence of psychopathology. These results are also relevant for clinicians and policy makers, underscoring the need for a family approach to psychopathology. The offspring of individuals with mental disorders is a group at greater odds of suicidality, and must therefore be actively screened for suicidal behaviors and psychopathology. As seen, adolescence is a critical stage, since the influence of parental mental disorders on offspring suicidality is most evident during this period, and suicide is the second most common cause of death in this age group [1]. Therefore, adolescence must be a major focus for interventions to prevent the onset and progression along the pathways to suicide.

## Supporting Information

S1 Dataset (XLS)
